# Functional Impairment and Its Determinants in Adolescents With Inherited Bleeding Disorders: A Single-Centre Cross-Sectional Study

**DOI:** 10.7759/cureus.117637

**Published:** 2026-10-08

**Authors:** Tayyaba Irfan, Moneeba Tahir, Valeed B Mansoor, Farwa Rasheed, Fibhaa Syed

**Affiliations:** 1 Internal Medicine, Pakistan Institute of Medical Sciences, Islamabad, PAK; 2 General Medicine, Pakistan Institute of Medical Sciences, Islamabad, PAK; 3 Medicine, Shaheed Zulfiqar Ali Bhutto Medical University, Islamabad, Islamabad, PAK

**Keywords:** health-related quality of life, health system in pakistan, people with haemophilia (pwh), platelet function, β-thalassemia

## Abstract

Background: Inherited bleeding disorders (IBDs) require long-term management through adolescence, a period of rapid social and educational development. This study set out to quantify day-to-day functional impairment in adolescents with IBDs attending a tertiary care centre in Islamabad, Pakistan, and to identify the characteristics associated with it.

Objective: This study aimed to determine the frequency and severity of functional impairment among adolescents with IBDs attending a tertiary care centre and to examine its association with demographic and clinical characteristics.

Methods: A cross-sectional study was conducted in the Department of Medicine, Pakistan Institute of Medical Sciences (PIMS), Islamabad, between June and September 2025. One hundred and fifty adolescents aged 8-19 years with a confirmed IBD were recruited by consecutive non-probability sampling. Patients who had undergone surgery within the preceding two months or who had an active bacterial infection, malignancy, hepatic impairment, or renal impairment were excluded. Functional impairment was measured using the Columbia Impairment Scale - Youth Version (CIS-Y), a 13-item instrument yielding a total score of 0-52. Scores were classified as minimal or mild (<15), moderate (15-30), and severe (>30). Associations were examined with the Pearson chi-square test, supplemented by Fisher’s exact test and an odds ratio for the comparison by sex, with p ≤ 0.05 taken as significant.

Results: The mean age was 13.93 ± 3.46 years, and 94 participants (62.7%) were male. Haemophilia A or B was the most common diagnosis (61, 40.7%), followed by platelet function disorders (38, 25.3%), von Willebrand disease (36, 24.0%), and other rare factor deficiencies (15, 10.0%). The mean CIS-Y score was 16.85 ± 11.35 (median 16.0, interquartile range 8.0-24.0). Sixty-six participants (44.0%) had minimal or mild impairment, 73 (48.7%) had moderate impairment, and 11 (7.3%) had severe impairment. Female sex was the only characteristic significantly associated with impairment severity (p = 0.015): 39 of 56 girls (69.6%) scored in the moderate or severe range compared with 45 of 94 boys (47.9%), giving an odds ratio of 2.50 (95% CI 1.24-5.02, Fisher’s exact p = 0.011). The median CIS-Y score was correspondingly higher in girls than boys (21.0 vs 14.0, p = 0.004). Age group, body mass index, socioeconomic status, residence, family structure, ethnicity, and type of bleeding disorder showed no significant association.

Conclusions: Close to half of the adolescents attending this centre with an IBD had moderate functional impairment, and girls had roughly twice the odds of impairment compared with boys. Severe impairment was uncommon. Functional and psychosocial assessment deserves a routine place in the follow-up of these patients, with particular attention to adolescent girls.

## Introduction

Inherited bleeding disorders (IBDs) arise from quantitative or qualitative defects in coagulation factors, platelets, or vascular integrity, and share a tendency to bleed excessively after minor trauma or surgery, or with no provocation at all. Haemophilia A and B, von Willebrand disease (VWD), platelet function defects, and the rarer single-factor deficiencies account for the majority of patients seen in clinical practice [1].

Pakistan has no national bleeding disorder registry, and published figures almost certainly understate the true burden. Consanguineous marriage is common, which raises the expected frequency of autosomal recessive conditions such as factor V and factor VII deficiency relative to Western series. In a hospital-based Pakistani series, inherited disorders accounted for close to a third of all patients presenting with a bleeding tendency [2]. Diagnostic capacity remains concentrated in a small number of tertiary centres, and many patients reach specialist care late or not at all [3].

The consequences for an adolescent extend well beyond the bleeding episode. Restriction of physical activity, interrupted schooling, uncertainty about when the next bleed will occur, and the visible marks of joint disease all arrive during the years in which peer relationships, independence, and self-image are being formed. Studies in adults and children with a range of bleeding phenotypes have documented reduced health-related quality of life, and this holds even where the underlying defect is mild or has never been fully characterised [4,5]. Psychological morbidity is also over-represented: in a South African cohort, adults with IBDs showed a higher burden of mental health disorders alongside poorer quality of life scores [6].

Health-related quality of life and functional impairment are related but not interchangeable. Quality of life instruments capture a person’s appraisal of their wellbeing across several domains, while impairment scales ask a narrower and more behavioural question about how much difficulty the person has in getting through everyday activities and relationships. Kamil and Mohammad used the latter approach in adolescents with inherited blood disorders and found that 43.6% had minimal to mild impairment, 46.8% moderate impairment, and 9.6% severe impairment [7]. The instrument they used, the Columbia Impairment Scale (CIS), was developed as a brief global measure of functioning in children and adolescents and is not tied to any single diagnosis [8], which makes it usable across a mixed bleeding disorder cohort where no disease-specific tool would apply to every patient [9].

Most of the Pakistani literature on IBDs is descriptive and haematological, addressing the spectrum of disease, factor levels, and diagnostic yield. The day-to-day functional cost to young patients has not been quantified locally, and internationally the evidence is skewed towards haemophilia in boys, leaving platelet function disorders, rare factor deficiencies, and adolescent girls comparatively unstudied. This study was therefore undertaken to measure the frequency and severity of functional impairment in a consecutive cohort of adolescents with IBDs at a tertiary centre in Islamabad and to identify the demographic and clinical characteristics associated with it.

## Materials and methods

Study design and setting

This cross-sectional study was conducted in the Department of Medicine, Pakistan Institute of Medical Sciences (PIMS), Islamabad, between June 2025 and September 2025. PIMS is a federal tertiary care hospital that receives referrals from Islamabad, Rawalpindi, and the surrounding districts, as well as from Khyber Pakhtunkhwa, Azad Jammu and Kashmir, and northern Punjab. The report follows the STROBE (STrengthening the Reporting of OBservational studies in Epidemiology) recommendations for cross-sectional studies.

Ethical considerations

The study was approved by the Ethics Research Review Committee (ERRC) of the PIMS, Islamabad (reference F-5-2/2024(ERRC)/PIMS, dated April 14, 2025). Approval was granted under the protocol title “Quality of Life Among Adolescents and Teenagers With Inherited Bleeding Disorders”; the present title reflects the instrument actually administered, and the study design, participants, and analysis are unchanged from the approved protocol. Consent was recorded on a single English-language document but verbally explained in Urdu. The document set out the nature of the underlying condition, explained that participation involved answering a set of questions as part of a research study, and recorded an assurance of confidentiality. Participants aged 18 years and above signed the document themselves as their own written informed consent. For participants younger than 18 years, the document was signed by a parent or legal guardian as written informed consent given on the participant’s behalf, along with verbal assent, with the guardian’s name and contact number recorded alongside the participant’s registration number. Participation was voluntary, no incentive was offered, and refusal carried no consequence for clinical care. Data were recorded against a study number without personal identifiers, and access was restricted to the investigators.

Participants

Adolescents of either sex aged 8-19 years with a previously confirmed diagnosis of an inherited bleeding disorder, documented in the hospital record, were eligible. Patients were excluded if they had undergone surgery within the preceding two months; had a bacterial infection confirmed clinically by fever together with a positive blood or urine culture; had a malignancy of any type; had impaired liver function, defined as a prothrombin activity below 75% of normal attributable to deficiency of vitamin K-dependent factors; or had impaired renal function, defined as a glomerular filtration rate below 60 mL/min/1.73 m². These exclusions were applied so that acute illness or acquired coagulopathy would not confound the assessment of functioning attributable to the underlying inherited disorder.

Recruitment was by consecutive non-probability sampling from the medical outpatient department. Every patient meeting the criteria during the study period was approached in order of presentation until the target sample was reached.

Sample size

The sample size was calculated for estimation of a population proportion with specified absolute precision, using a 95% confidence level, an anticipated proportion of severe functional impairment of 9.6% taken from Kamil and Mohammad [7], and an absolute precision of 4.8%. This yielded a required sample of 145, which was rounded up to 150 to allow for incomplete responses.

Measurement of functional impairment

Functional impairment was assessed with the Columbia Impairment Scale - Youth Version (CIS-Y) [8,9]. The CIS-Y is a copyrighted instrument developed by Bird and colleagues and is reproduced here only in summary; the full item set is available from the original publication [8]. It contains 13 items grouped into four domains: interpersonal relations (relationships with mother or mother figure, father or father figure, siblings, peers of the same age, and adults other than parents), psychopathology (feeling unhappy or sad, feeling nervous or afraid, and getting into trouble), school or work functioning (behaviour at school or work, and schoolwork or job performance), and leisure and home functioning (having fun, involvement in activities such as sports or hobbies, and behaviour at home). Each item is scored from 0 (“no problem”) to 4 (“a very bad problem”), with an additional “not applicable or don’t know” option that is treated as a skip and is not carried into the total. Item scores are summed to give a total between 0 and 52, with higher scores indicating greater impairment. Internal consistency reported for the youth version in previous samples has ranged from 0.69 to 0.84. The CIS-Y is distributed as a freely available measurement protocol through the PhenX Toolkit (RTI International, Research Triangle Park, North Carolina), which states that the protocol is freely available and that permission is not required for its use [9,10]. No licence fee was payable, and no separate written permission was required for use of the instrument in this study.

A total score of 15 or above was taken to indicate functional impairment. Consistent with the categorisation used by Kamil and Mohammad [7], scores were grouped as minimal or mild impairment (<15), moderate impairment (15-30), and severe impairment (>30).

The CIS is designed for completion either by the young person or by a parent or caregiver reporting on the young person's behalf, and the instruction stem on the administered form is worded to accommodate either informant [9]. In the present study, the scale was answered by the adolescent directly in 120 cases (80.0%). In the remaining 30 cases (20.0%), an accompanying parent or guardian answered on the participant's behalf, where the participant was below 10 years of age or was unable or unwilling to respond independently. All proxy interviews were conducted with a parent or guardian who lived with the participant and had accompanied them to the clinic.

Data collection

A structured proforma developed by the investigators for this study was used to record age and age group, sex, height and weight for calculation of body mass index, monthly household income in Pakistani rupees as a proxy for socioeconomic status, urban or rural residence, family structure, self-reported ethnicity, the documented type of inherited bleeding disorder, and the CIS-Y total score with its resulting impairment category. The proforma is reproduced in full in the Appendices. Clinical history was taken and physical examination performed by the trainee researcher for all participants, and the diagnosis recorded on the proforma was verified against the hospital laboratory and clinical record rather than taken from patient report.

Statistical analysis

Data were entered and analysed in IBM SPSS Statistics for Windows, Version 25 (Released 2017; IBM Corp., Armonk, New York). Continuous variables are summarised as mean ± standard deviation and as median with interquartile range; categorical variables are summarised as frequency and percentage. CIS-Y total scores departed from normality on the Shapiro-Wilk test (W = 0.952, p < 0.001), so between-group comparisons of the score used the Mann-Whitney U test.

The association between CIS-Y impairment category and each demographic and clinical characteristic was tested with Pearson's chi-square test, with the chi-square statistic, degrees of freedom, and exact p-value reported for each comparison in Table 5. At least one cell in each of these tables had an expected count below five, so these p-values are approximate. Because sex was the only characteristic reaching significance, that comparison was repeated after collapsing the moderate and severe categories into a single impaired category; the resulting two-by-two table was tested with Fisher's exact test and summarised with an odds ratio and 95% confidence interval. Fisher's exact test was applied to this two-by-two table only. Every p-value reported in Table 5 is from Pearson's chi-square test. A p-value of 0.05 or less was taken as significant. No adjustment was made for multiple comparisons, and the subgroup analyses are accordingly regarded as hypothesis-generating.

## Results

Participant characteristics

One hundred and fifty adolescents were enrolled. The mean age was 13.93 ± 3.46 years, with 89 participants (59.3%) in the 13-19-year age group and 61 (40.7%) aged 8-12 years. Ninety-four participants (62.7%) were male. BMI was within the 18-25 kg/m² band in 132 cases (88.0%), with a mean of 21.89 ± 2.63 kg/m².

Sixty-nine participants (46.0%) came from households reporting a monthly income below PKR 50,000. Eighty-seven (58.0%) lived in urban areas, and 99 (66.0%) lived in a combined family structure. Punjabi was the most common ethnic background (73, 48.7%), followed by Pashtun (24, 16.0%), Sindhi and Kashmiri (20 each, 13.3%), and Balochi (13, 8.7%). Full demographic details appear in Table 1.

**Table 1:** Demographic characteristics of study participants (n = 150)

Variable	Category	n (%)
Age group	8-12 years	61 (40.7)
	13-19 years	89 (59.3)
Sex	Male	94 (62.7)
	Female	56 (37.3)
Body mass index	18-25 kg/m²	132 (88.0)
	26-30 kg/m²	18 (12.0)
Monthly household income	<50,000 PKR	69 (46.0)
	50,000-100,000 PKR	57 (38.0)
	>100,000-300,000 PKR	24 (16.0)
Residence	Rural	63 (42.0)
	Urban	87 (58.0)
Family structure	Combined family	99 (66.0)
	Separate house	37 (24.7)
	Separate flat	14 (9.3)
Ethnicity	Punjabi	73 (48.7)
	Pashtun	24 (16.0)
	Sindhi	20 (13.3)
	Kashmiri	20 (13.3)
	Balochi	13 (8.7)

Distribution of bleeding disorders

Haemophilia A or B was the most common diagnosis, present in 61 participants (40.7%). Platelet function disorders accounted for 38 cases (25.3%) and vWD for 36 (24.0%). The remaining 15 participants (10.0%) had rare inherited single-factor deficiencies, comprising factor V and factor VII deficiency (Table 2).

**Table 2:** Distribution of inherited bleeding disorders (n = 150)

Bleeding disorder	n	%
Haemophilia A or B	61	40.7
Platelet function disorder	38	25.3
von Willebrand disease	36	24.0
Other rare factor deficiencies (factor V, factor VII)	15	10.0
Total	150	100.0

Functional impairment

The mean CIS-Y total score was 16.85 ± 11.35, with a median of 16.0 (interquartile range 8.0-24.0) and a range of 0 to 50. Eighty-four participants (56.0%) scored at or above the impairment threshold of 15. Within the three-band classification, 66 participants (44.0%) had minimal or mild impairment, 73 (48.7%) had moderate impairment, and 11 (7.3%) had severe impairment (Tables 3, 4). 

**Table 3:** Distribution of functional impairment (n = 150)

Category	n	%
Minimal or mild impairment (CIS-Y < 15)	66	44
Moderate impairment (CIS-Y 15–30)	73	48.7
Severe impairment (CIS-Y > 30)	11	7.3

**Table 4:** Descriptive statistics of study variables (n = 150)

Variable	Mean ± SD	Median (IQR)	Minimum	Maximum
Age (years)	13.93 ± 3.46	14.0 (11.0–17.0)	8	19
Body mass index (kg/m²)	21.89 ± 2.63	21.6 (19.6–23.6)	18	28.5
CIS-Y total score	16.85 ± 11.35	16.0 (8.0–24.0)	0	50

Factors associated with impairment

Sex was the only characteristic significantly associated with impairment category (χ² = 8.352, df = 2, p = 0.015). With the moderate and severe categories collapsed, 39 of 56 girls (69.6%) and 45 of 94 boys (47.9%) fell in the impaired range; Fisher's exact test on this two-by-two table gave p = 0.011, with an odds ratio for female sex of 2.50 (95% CI 1.24-5.02). All expected counts in the two-by-two table exceeded five, so an exact test was not strictly required, and the corresponding Pearson chi-square result was consistent (χ² = 6.750, df = 1, p = 0.009). The difference was also present at the score level: the median CIS-Y total score was 21.0 (interquartile range 11.0-27.0) in girls and 14.0 (interquartile range 6.0-22.0) in boys (Mann-Whitney U = 1894.5, Z = -2.867, p = 0.004).

No other variable reached significance. Moderate impairment was slightly more common in the younger age group (32 of 61, 52.5%) than in the older group (41 of 89, 46.1%), but the difference was not significant (p = 0.741). Ethnicity showed the widest spread of proportions, with severe impairment recorded in 3 of 13 Balochi participants (23.1%) and in none of the 24 Pashtun participants, and came closest to significance without reaching it (p = 0.082). Body mass index, household income, residence, and family structure showed no association. Type of bleeding disorder was likewise not significantly associated with impairment (p = 0.558), although severe impairment was proportionally most common in the rare factor deficiency group (3 of 15, 20.0%) and least common in VWD (1 of 36, 2.8%). These findings are set out in full in Table 5. 

**Table 5:** Association of participant characteristics with CIS-Y impairment category (n = 150) All p-values in this table are from Pearson’s chi-square test. Fisher’s exact test was not applied to any comparison in this table. Percentages are row percentages within each category and may not total 100 because of rounding. At least one cell in each contingency table had an expected count below five, so these p-values are approximate. The minimum expected counts were 4.47, 4.11, 1.32, 1.76, 4.62, 1.03, 0.95, and 1.10 for the eight comparisons in the order listed. No adjustment was made for multiple comparisons; the subgroup analyses are regarded as hypothesis-generating. The separate two-by-two analysis of sex, in which the moderate and severe categories were collapsed into a single impaired category, is reported in the text (Fisher’s exact p = 0.011; odds ratio for female sex 2.50, 95% CI 1.24-5.02). CIS-Y: Columbia Impairment Scale - Youth Version; BMI: body mass index; PKR: Pakistani rupees; χ²: chi-square statistic; df: degrees of freedom; CI: confidence interval.

Variable	Category	n	CIS-Y impairment category, n (%)			χ²	df	p-value
			Minimal or mild	Moderate	Severe			
Age group	8-12 years	61	25 (41.0)	32 (52.5)	4 (6.6)	0.601	2	0.741
	13-19 years	89	41 (46.1)	41 (46.1)	7 (7.9)			
Sex	Male	94	49 (52.1)	41 (43.6)	4 (4.3)	8.352	2	0.015
	Female	56	17 (30.4)	32 (57.1)	7 (12.5)			
BMI category	18-25 kg/m²	132	56 (42.4)	65 (49.2)	11 (8.3)	2.196	2	0.334
	26-30 kg/m²	18	10 (55.6)	8 (44.4)	0 (0.0)			
Monthly household income	<50,000 PKR	69	30 (43.5)	33 (47.8)	6 (8.7)	3.329	4	0.504
	50,000-100,000 PKR	57	28 (49.1)	27 (47.4)	2 (3.5)			
	>100,000-300,000 PKR	24	8 (33.3)	13 (54.2)	3 (12.5)			
Residence	Rural	63	24 (38.1)	33 (52.4)	6 (9.5)	1.879	2	0.391
	Urban	87	42 (48.3)	40 (46.0)	5 (5.7)			
Family structure	Combined family	99	42 (42.4)	51 (51.5)	6 (6.1)	2.144	4	0.709
	Separate house	37	16 (43.2)	17 (45.9)	4 (10.8)			
	Separate flat	14	8 (57.1)	5 (35.7)	1 (7.1)			
Ethnicity	Punjabi	73	39 (53.4)	29 (39.7)	5 (6.8)	13.992	8	0.082
	Sindhi	20	6 (30.0)	13 (65.0)	1 (5.0)			
	Pashtoon	24	8 (33.3)	16 (66.7)	0 (0.0)			
	Balochi	13	4 (30.8)	6 (46.2)	3 (23.1)			
	Kashmiri	20	9 (45.0)	9 (45.0)	2 (10.0)			
Type of bleeding disorder	Haemophilia A or B	61	27 (44.3)	30 (49.2)	4 (6.6)	4.887	6	0.558
	von Willebrand disease	36	17 (47.2)	18 (50.0)	1 (2.8)			
	Platelet function disorder	38	17 (44.7)	18 (47.4)	3 (7.9)			
	Rare factor deficiency	15	5 (33.3)	7 (46.7)	3 (20.0)			

## Discussion

Just over half of the adolescents in this cohort scored at or above the CIS-Y impairment threshold, and the impairment was predominantly moderate rather than severe. Of the eight demographic and clinical characteristics examined, only sex was associated with the severity of impairment, with girls carrying roughly twice the odds of impairment compared with boys.

The distribution we observed is close to that reported by Kamil and Mohammad in adolescents with inherited blood disorders, who found 43.6% with minimal to mild impairment, 46.8% with moderate impairment, and 9.6% with severe impairment [7], against our figures of 44.0%, 48.7%, and 7.3%. Two cohorts in different health systems, assessed with the same instrument, arriving at nearly the same distribution is a reasonable indication that the pattern is a property of the population rather than of local circumstances. It also suggests that the CIS-Y behaves consistently in adolescents whose primary problem is haematological rather than psychiatric, a setting some distance from the one in which the scale was developed.

That moderate impairment predominates while severe impairment remains uncommon fits with work showing that young people with hereditary bleeding disorders are not uniformly disadvantaged. Neuner and colleagues compared children and adolescents with hereditary bleeding disorders against siblings and peers and found health-related quality of life broadly comparable, with no consistent deficit across the major domains [11]. Our data are compatible with that picture, provided the difference in what is being measured is kept in view: the CIS-Y asks how much difficulty the young person is having, not how they appraise their life overall, and the two questions can give different answers in the same patient.

The sex difference is the most substantial finding here and also the one most in need of careful interpretation. It was present both in the categorical classification and in the underlying continuous score, which makes it unlikely to be an artefact of where the impairment cut-offs happen to fall. Mokhtar and colleagues, studying girls and adolescent females with bleeding symptoms, described a pattern of delayed diagnosis, greater symptom burden and fatigue, and poorer quality of life outcomes relative to males [12]. The direction of our result matches theirs. The obvious mechanism is menstruation: heavy menstrual bleeding is often the presenting and the dominant symptom in adolescent girls with VWD, platelet function disorders, and rare factor deficiencies, and it recurs on a schedule that no amount of activity modification can avoid. Cultural factors plausibly compound this, since menstrual symptoms are not readily discussed and may not be brought to medical attention promptly. We must be explicit, however, that we did not collect menstrual history, age at menarche, or any measure of menstrual blood loss. The menstrual explanation is therefore a hypothesis consistent with our data and with the published literature, not a finding of this study. Establishing it would require a cohort in which menstrual variables are recorded and modelled directly.

Adolescence itself adds a layer that is independent of the bleeding phenotype. Königs and colleagues have argued that teenagers with haemophilia require a distinct clinical approach, describing deteriorating adherence to prophylaxis as young people seek acceptance among peers and attempt to minimise the visible constraints of their condition, with anxiety and reduced participation following [13]. Related work by the same group on self-esteem in adolescents with inherited blood disorders points in the same direction [14]. Nearly half of our cohort scoring in the moderate band, without a corresponding excess of severe impairment, is what one would expect if the dominant burden is this kind of persistent low-grade interference with ordinary activity rather than catastrophic disability.

We found no significant association between the type of bleeding disorder and impairment (p = 0.621), though the proportions differed between groups. Severe impairment was proportionally most common among the rare factor deficiencies (3 of 15, 20.0%) and least common in VWD (1 of 36, 2.8%). Goudemand and colleagues reported that health-related quality of life in children with VWD declines as von Willebrand factor levels fall [15], which would predict a spread of outcomes within our VWD subgroup rather than uniformly low impairment. We cannot say why our VWD patients did comparatively well, because we did not record factor levels, bleeding phenotype, or treatment regimen. Milder disease expression among those attending our clinic is one possibility; effective management is another; small subgroup size and chance are a third and should not be dismissed. With only 15 patients in the rare deficiency group, the 20% severe impairment figure rests on three individuals and cannot support any inference.

Findings from comparable settings reinforce the general direction. Ahmed and colleagues found that bleeding frequency, joint involvement, and emotional distress all shaped quality of life in Egyptian children with haemophilia [16]. Dsouza and colleagues, reporting on Indian children and adolescents with haemophilia, described substantial psychosocial and family-related difficulty, with scores tending to worsen with age although the difference between older and younger participants did not reach significance [17]. Our own age comparison was similarly non-significant. One reading is that the increasing autonomy of later adolescence raises functional demands at the same rate that accumulated experience improves coping, leaving no net difference detectable in a cross-sectional sample of this size.

The absence of any socioeconomic or residential gradient deserves comment, since such gradients are the rule in most chronic disease research. A likely explanation is selection: all our participants had already reached and were being followed at a federal tertiary centre, which removes much of the variation in access that would otherwise separate a rural low-income family from an urban higher-income one. Adolescents who never reach such a centre are, by definition, absent from this cohort, and they are the ones in whom a socioeconomic effect would most likely be visible.

The choice of instrument reflects a recognised problem in this field. Huisman and colleagues, in a scoping review of health-related quality of life research in children with bleeding disorders, documented wide variation in the instruments used and identified specific evidence gaps around platelet function disorders, young females, and non-haemophilia diagnoses [18]. No disease-specific instrument covers haemophilia, VWD, platelet function disorders, and rare factor deficiencies simultaneously, so a mixed cohort forces a generic measure. The CIS-Y served that purpose, and its use here extends the available data into exactly the subgroups the review identified as under-studied. The cost is that a generic scale is insensitive to the particular concerns of bleeding disorders, such as joint pain, infusion burden, or menstrual disruption, none of which appear in the 13 items.

This study has several limitations. The cross-sectional design establishes association only, and the direction of any relationship between sex and impairment cannot be established from these data. We collected no measures of disease severity: factor levels, annual bleeding rate, joint health scores, and prophylaxis status were all unavailable, and each is a plausible confounder of the associations reported. Recruitment was consecutive but non-probability, from a single tertiary centre, so the cohort is likely to over-represent patients with established diagnoses and reasonable access to care. For participants under 18 years, written informed consent was obtained from a parent or legal guardian, but the participants’ own assent was not separately documented; 61 participants were aged 8-12 years, and documented assent would have strengthened the ethical record. Responses were self-reported by the adolescent in 80% of cases and given by a parent or guardian in the remaining 20%; youth and caregiver reports on the CIS have not been shown to be interchangeable, and this mixture introduces measurement heterogeneity that we could not quantify or adjust for. Only CIS-Y total scores were retained in the study database, so internal consistency of the 13 items within this sample could not be assessed and no sample-specific reliability coefficient can be reported; we rely instead on the psychometric properties established for the scale elsewhere [9,10]. Relatedly, the “not applicable” option is excluded from the CIS-Y total, so participants who skipped items receive systematically lower totals, which may have shifted some cases into a milder category, and the number of such responses could not be recovered. Eight comparisons were performed without adjustment for multiplicity, so the ethnicity result in particular (p = 0.090) should not be read as a near-miss but simply as uninformative. Finally, subgroup sizes for the rarer disorders were too small to support meaningful comparison.

Against this, the study covers the full range of inherited bleeding disorders in a single consecutive cohort rather than restricting itself to haemophilia, uses a validated instrument with published psychometric properties, and reports exact tests appropriate to the sparse contingency tables involved. Comparable local data did not previously exist.

The practical implication is straightforward. Functional assessment is not currently part of routine bleeding disorder follow-up in most Pakistani centres, and a 13-item scale that takes a few minutes to administer would identify the substantial minority of adolescents who are struggling. Adolescent girls warrant particular attention, and the assessment should include menstrual history, which is the most likely modifiable contributor to their excess impairment. Future work should record disease severity and treatment variables, follow patients longitudinally through the transition to adult care, and test whether targeted intervention, whether optimised prophylaxis, menstrual management, or psychological support, moves these scores.

## Conclusions

Moderate functional impairment was present in almost half of the adolescents attending this tertiary centre with an IBD, while severe impairment was uncommon. Female sex was the only characteristic significantly associated with greater impairment, with girls showing approximately twice the odds of moderate or severe impairment compared with boys. Age, body mass index, socioeconomic status, residence, family structure, ethnicity, and type of bleeding disorder showed no significant association. These findings support incorporating brief functional assessment into routine follow-up for adolescents with inherited bleeding disorders and give particular reason to examine the needs of adolescent girls, in whom menstrual bleeding is a plausible but as yet untested explanation for the excess impairment observed.

## Appendices

Appendix 1. Study data collection proforma

The following proforma was developed by the investigators for this study and completed for every participant at the time of enrolment.

**Table 6:** Study data collection proforma

Field	Entry format
Patient number	Free text
Date	Free text
Registration number	Free text
Patient name	Free text
Age	Years
Age group	Adolescent / Teenage
Monthly household income (PKR)	< 50,000 / 50,000-100,000 / > 100,000-300,000
Body mass index	kg/m²
Sex	Male / Female
Residential status	Rural / Urban
Family structure	Combined family / Separate in house / Separate in flat
Ethnic group	Punjabi / Sindhi / Pashtun / Balochi / Kashmiri
Type of bleeding disorder	Free text, verified against clinical and laboratory record
Columbia Impairment Scale (Youth Version) total score	0-52
Functional impairment category	Minimal or mild / Moderate / Severe

Appendix 2. Assessment instrument

Functional impairment was measured using the Columbia Impairment Scale - Youth Version, a 13-item instrument developed by Bird et al. The scale is copyrighted by its developers and is therefore not reproduced here. Item content is summarised by domain in the Materials and Methods section, and the full instrument is available from the original publication [8] and from the psychometric evaluation by Attell et al. [9].

Appendix 3. Consent documentation

Consent was recorded on a single-page bilingual document presenting identical text in English and Urdu. The document confirmed that the treating doctor had explained the participant’s condition, that associated problems required evaluation for better management, that a small number of questions would be asked as part of a research study, that the participant or guardian was willing to take part, and that confidentiality of personal details had been assured. Fields were provided for date, registration number, participant name, signature with the guardian’s name entered where a guardian signed on the participant’s behalf, and a contact number. The single document therefore served as the participant’s own informed consent where the participant was of age to give it, and as guardian informed consent for participants below 18 years. The document is not reproduced in full as it contains no additional methodological information.
